# Identification of a High-Yield and Low-Cadmium-Accumulating Rice Cultivar by LAMP-Based *Gn1a-i* Screening and Physiological Evaluation

**DOI:** 10.3390/genes17040482

**Published:** 2026-04-18

**Authors:** Xiyi Chen, Shangdu Zhang, Yaoxian Chin, Mingshi Lao, Guibo Zhang, Fengtao Yu, Linfeng Cheng, Yonghang Tian

**Affiliations:** 1 College of Food Science and Engineering, Hainan Tropical Ocean University, No. 1 Yucai Road, Sanya 572022, China; mt148713651@163.com (X.C.); mingshilao@gmail.com (M.L.); m15004242440@163.com (G.Z.); 13086072117@163.com (F.Y.); 13178903829@163.com (L.C.); 2 Rice Research Institute of Guizhou Province, Guizhou Academy of Agricultural Sciences, Guiyang 550025, China; zsd1411@163.com; 3 Yazhou Bay Innovation Institute, College of Food Science and Engineering, Hainan Tropical Ocean University, No. 1 Yucai Road, Sanya 572022, China; chinyx1@hntou.edu.cn; 4 HNTOU-UM Marine Biotechnology Joint Research Laboratory, Hainan Tropical Ocean University, No. 1 Yucai Road, Sanya 572022, China

**Keywords:** *Oryza sativa* L., loop-mediated isothermal amplification, variety screening, *Gn1a* gene, cadmium content

## Abstract

Background/Objectives: With the acceleration of global industrialization and continuous population growth, the world is increasingly confronted with the dual challenges of food insecurity and cultivated land contamination. The screening and breeding of rice varieties with superior agronomic traits and low heavy metal accumulation have therefore become important strategies for ensuring food safety and sustainable agricultural production. Methods: In this study, rice varieties carrying the *Gn1a-i* gene and exhibiting specific cadmium (Cd) accumulation characteristics were screened using a combination of molecular marker detection and cadmium accumulation evaluation. Specific loop-mediated isothermal amplification (LAMP) primers targeting the *Gn1a-i* gene were designed and combined with a lateral flow dipstick (LFD) assay to enable rapid genetic screening of rice varieties. A six-day hydroponic experiment under cadmium stress was conducted across three temperature ranges (15–20 °C, 22–27 °C, and 30–35 °C), and cadmium accumulation in different plant organs (roots, stem sheath, and leaves) was analyzed. Results: Seven varieties carrying the *Gn1a-i* gene, including Xiangwanxian 12, were identified among ten tested rice varieties. Xiangwanxian 12 was subsequently selected for further evaluation, with the high-cadmium-accumulating variety Yuzhenxiang used as a control. At 144 h, the total Cd content in the measured organs of Xiangwanxian 12 was 9.6%, 4.0%, and 23.2% lower than that of Yuzhenxiang under low, medium, and high temperatures, respectively (one-tailed *t*-test, *p* < 0.01 for all three temperatures). Conclusions: The integration of LAMP-based genotyping and physiological evaluation provides a novel and reliable strategy for identifying low-Cd rice germplasm. Xiangwanxian 12, which carries the *Gn1a-i* allele and exhibits consistently lower Cd accumulation than Yuzhenxiang, suggests potential as a candidate for breeding high-yield, low-Cd rice cultivars.

## 1. Introduction

Food security refers to ensuring that all individuals have continuous access to sufficient, nutritious, and safe food necessary for maintaining health and well-being [1]. Ensuring an adequate and safe food supply has long been a fundamental basis for social stability and economic development and remains a key global concern for the international community [2]. Rice (*Oryza sativa* L.), a cereal crop belonging to the genus *Oryza* in the family Poaceae, is one of the most important staple foods worldwide and provides the primary dietary energy source for more than two-thirds of the global population. Therefore, maintaining both the productivity and safety of rice is essential for safeguarding global food security [3,4].

However, with the acceleration of global industrialization and continuous population growth, soil contamination by heavy metals has become a concerning issue in agriculture [5,6,7]. Cadmium is a highly mobile heavy metal in soils and can be readily absorbed by rice roots in contaminated paddy fields. Bioavailable forms of cadmium in soil is primarily taken up by rice roots via transport systems for essential divalent cations, such as manganese (Mn) and zinc (Zn) [8]. Once absorbed, a portion of Cd is immobilized through adsorption to root cell walls or sequestration into vacuoles, while the remainder is loaded into the xylem and translocated to aerial organs, including stems and leaves. During the grain-filling stage, Cd accumulated in vegetative tissues can be further remobilized via the phloem and ultimately deposited in the grains [9]. Consequently, Cd may enter the human body through the food chain, posing significant health risks, including reproductive and developmental toxicity, neurotoxicity, and carcinogenic effects. Due to its preferential accumulation in the kidneys and bones, chronic Cd exposure can result in kidney dysfunction, cancer, cardiovascular diseases, and osteoporosis [10]. At the same time, the increasing demand for rice, driven by population growth and rising living standards, has placed a higher emphasis on crop yield. Consequently, the development and screening of rice varieties that combine high yield potential with low cadmium accumulation has become an important strategy to address the dual challenges of food production and food safety.

However, a trade-off often exists between yield potential and stress tolerance in crops [11]. As such, rice varieties with low cadmium accumulation frequently exhibit reduced growth vigor and lower yield [12]. Nevertheless, only a limited number of genes have been identified that can break this limitation and achieve simultaneous improvement in both yield and stress tolerance [13,14]. The *Gn1a* gene encodes cytokinin oxidase/dehydrogenase (*OsCKX2*), which regulates cytokinin levels in young panicle primordia and thereby influences spikelet development [15]. A specific allele of this gene, *Gn1a-i*, contains a 16 bp deletion in the 5′ untranslated region (5′UTR) of its promoter which reduces the expression level of *Gn1a*, leading to increased cytokinin accumulation. Since cytokinins positively regulate shoot apical meristem activity, this genetic variation would increase the number of branches and grains per panicle, ultimately enhancing rice yield [16]. Therefore, identifying and obtaining rice germplasm that carries the *Gn1a-i* allele while exhibiting low cadmium accumulation is of great significance for breeding of safe, high-yielding rice cultivars. Despite extensive studies on the genetic basis of grain number and on cadmium accumulation in rice, few efforts have integrated high-yield allele screening with physiological evaluation of heavy metal uptake [17,18].

Loop-mediated isothermal amplification (LAMP) is an isothermal amplification technique that employs *Bst* DNA polymerase with strand displacement activity. Using two pairs of specific primers (F3/B3 and FIP/BIP) targeting six specific regions of the target sequence, LAMP enables rapid isothermal amplification of the template nucleic acids. Compared with conventional polymerase chain reaction (PCR), LAMP offers advantages such as a lower detection limit, higher sensitivity, better specificity, shorter reaction time, and greater operational convenience [19,20].

The present study aims to develop a set of LAMP molecular markers that targets the 16 bp deletion in the 5′UTR of the *Gn1a-i* allele to enable rapid screening of rice varieties carrying this high-yield allele [17,21,22]. The reliability of the LAMP markers will be validated using conventional PCR. Subsequently, rice varieties identified as carrying the *Gn1a-i* allele will be evaluated for cadmium accumulation characteristics. Through this integrated approach, the aim is to screen for rice varieties possessing both high yield potential and low cadmium accumulation. The findings of this study are expected to contribute to increased rice production in contaminated agricultural areas while reducing cadmium levels in rice grains, thereby enhancing food safety. In addition, the data generated in the present work will provide valuable germplasm resources for breeding programs aimed at addressing global food security challenges.

## 2. Materials and Methods

### 2.1. Plant Material

The experimental materials used in this study comprised eleven rice varieties, including Asian cultivated rice (*Oryza sativa* L.) and African cultivated rice (*Oryza glaberrima* Steud.). The *O. sativa* varieties included Xiangwanxian 12, 9311, Zhenshan 97, N22, R498, R600, Jiayu 253, and Yuzhenxiang (indica subspecies), as well as Zhonghua 11 and Nipponbare (japonica subspecies). The *O. glaberrima* variety CG14 was also included. All rice materials were cultivated and provided by the Rice Research Institute of Guizhou Province.

### 2.2. LAMP Primer Design

The target sequence for primer design was determined based on the DNA sequence of rice materials containing the *Gn1a-i* allele. Sequence alignment was performed using the NCBI nucleotide database (https://blast.ncbi.nlm.nih.gov/Blast.cgi (accessed on 20 March 2024)) to identify the 16 bp deletion located in the 5′ untranslated region (5′UTR) of the *Gn1a-i* allele and its conserved flanking regions. The LAMP primers (F3, B3, FIP, and BIP) were designed using PrimerExplorer V5 (http://primerexplorer.jp/lampv5e/index.html (accessed on 21 March 2024)) according to the six target regions required for LAMP. To ensure specificity for the *Gn1a-i* allele, part of the B1 region of the inner primer BIP was designed to overlap the 16 bp deletion site in the 5′UTR. The 5′ ends of primers F3 and FIP were subsequently labeled with 6-FAM and biotin, respectively. All primers were synthesized by Sangon Biotech Co., Ltd. (Shanghai, China).

### 2.3. PCR Primer Design

To validate the accuracy of the LAMP screening results, PCR primers targeting the insertion–deletion (InDel) marker specific to the *Gn1a-i* allele were designed using Primer3 (https://primer3.ut.ee/ (accessed on 15 April 2024)). Primer design was based on the 16 bp deletion site in the 5′UTR region of the *Gn1a* gene and its conserved upstream and downstream sequences. The primers were synthesized by Sangon Biotech Co., Ltd. (Shanghai, China).

### 2.4. Genomic DNA Extraction

Genomic DNA was extracted from rice leaf tissues using a Plant Genomic DNA Extraction Kit (DP305, Tiangen Biotech Co., Ltd., Beijing, China) following the manufacturer’s protocol based on the silica membrane spin column method. The extracted DNA was stored at −20 °C until further use [23].

### 2.5. LAMP Assay

LAMP is a highly sensitive technique capable of detecting very low levels of target nucleic acids, often down to a few copies [24]. It also exhibits high specificity due to the use of multiple primers recognizing several distinct regions of the target sequence. However, its high amplification efficiency increases the risk of carryover contamination, which may result in false-positive outcomes. Therefore, strict contamination control measures will be implemented throughout the study to minimize the risk of such false-positive results [21,25].

In this study, LAMP was performed to screen rice varieties carrying the *Gn1a-i* allele using Isothermal Amplification PCR Mixture (Universal Type) (B532455-0040, Sangon Biotech (Shanghai) Co., Ltd.). The reaction mixture (12.5 μL) contained 6.25 μL 2× LAMP Master Mix, 1 μL FIP, 1 μL BIP, 0.25 μL F3, 0.25 μL B3, 0.25 μL DNA polymerase, 0.5 μL genomic DNA template, and nuclease-free water to the final volume. The amplification reaction was conducted at 59.4 °C for 60 min, followed by enzyme inactivation at 85 °C for 10 min and incubation at 12 °C for 5 min. The resulting LAMP products (5 μL) were first analyzed by 3% agarose gel electrophoresis and visualized using a gel imaging system (ChemStudio, Jena Analysis Instruments Co., Ltd., Jena, Germany). The presence of a ladder-like band pattern indicated successful amplification of the target sequence.

For rapid detection, LAMP products were also analyzed using a lateral flow dipstick (LFD, 24TEX, Sangon Biotech (Shanghai) Co., Ltd., Shanghai, China). Briefly, 5 μL of amplification product was diluted 20-fold with ddH_2_O, and 80 μL of the diluted solution was applied to the LFD strip. Amplified products labeled with both 6-FAM and biotin formed complexes with colloidal gold-labeled anti-biotin antibodies and migrated along the nitrocellulose membrane. The complexes were captured by anti-6-FAM antibodies at the test line, producing a visible band, while excess conjugates were captured at the control line. The presence of both test and control lines indicated a positive result for the *Gn1a-i* allele [26,27]. Although LAMP-LFD assay result is qualitative and does not provide quantitative information on gene expression levels, it is sufficient for the purpose of allele screening in this study.

### 2.6. PCR Amplification

PCR amplification was performed to confirm the presence of the *Gn1a-i* allele in the tested rice varieties. The reaction mixture (10 μL) consisted of 5 μL Taq MasterMix II, 1 μL genomic DNA template, 1 μL forward primer (Gn1a-1F), 1 μL reverse primer (Gn1a-1R), and 2 μL ddH_2_O.

The PCR program (t100, Bio-Rad Laboratories, Inc., Hercules, CA, USA) was set as follows: an initial denaturation at 94 °C for 3 min, followed by 30 cycles of denaturation at 94 °C for 30 s, annealing at 57.4 °C for 30 s, and extension at 72 °C for 1 min. A final extension was performed at 72 °C for 5 min. After the reaction, PCR products (5 μL) were analyzed by 3% agarose gel electrophoresis, and the amplified fragment size was used to determine the presence of the *Gn1a-i* allele. The results were compared with those obtained from the LAMP assay to evaluate the accuracy of the LAMP screening method.

### 2.7. Evaluation of Cadmium Accumulation

Rice varieties identified as carrying the *Gn1a-i* allele were further evaluated for cadmium accumulation under hydroponic conditions. Rice seeds were surface-sterilized via soaking in 0.02% (*w*/*v*) trichloroisocyanuric acid, germinated, and grown in a controlled growth chamber (JC-150-GSIE, Qingdao Jingcheng Instruments, Shandong, China). When seedlings reached the three-leaf stage, uniformly growing plants were selected, placed in sponge, and transferred to planting baskets supported by foam boards. The cultivation board was then placed in a plastic box containing Kimura B nutrient solution (pH 5.5).

To simulate cadmium stress conditions, seedlings with uniform growth, evaluated in terms of plant height, tillering, and fresh weight, were transferred to nutrient solution containing 2 mg/L Cd prepared from analytical grade CdCl_2_·2.5H_2_O (analytical grade, Sinopharm Group, Shanghai, China) and cultivated for six days [28]. Three temperature treatments were applied in the growth chamber: low temperature (15–20 °C), medium temperature (22–27 °C), and high temperature (30–35 °C) [29]. The photoperiod was maintained at 14 h light (12,000 lx, 06:00–20:00) and 10 h dark (20:00–06:00).

Plant samples were collected as three biological replicates (three individual seedlings per variety per time point) at 24 h intervals (24, 48, 72, 96, 120, and 144 h) for each temperature treatment. Whole plants were separated into roots, stem sheath, and leaves using sterilized scissors. After washing with deionized water, samples were blotted dry and weighed to determine fresh weight. Tissues were then oven-dried at 105 °C for 30 min to inactivate enzymes and subsequently dried at 80 °C until constant weight were achieved to determine dry weight. The dried samples were ground and passed through a 100-mesh sieve.

Cadmium content was determined according to the method described in GB 5009.15-2023 National Food Safety Standard—Determination of Cadmium in Food. Briefly, dried and finely powdered samples (0.50 g) were weighed into microwave digestion vessels, followed by the addition of 5 mL of concentrated nitric acid (HNO_3_) and 5 mL of 30% hydrogen peroxide (H_2_O_2_). The samples were subjected to microwave digestion until complete mineralization was achieved. After digestion, the solution was heated at 140 °C to near dryness to remove excess acid. The digestion vessel was rinsed three times with 1% nitric acid, and the combined solution was transferred into a 25 mL volumetric flask and diluted to volume with 1% nitric acid. A reagent blank was prepared using the same procedure. Cadmium concentrations in the digested solutions were determined using an atomic absorption spectrophotometer (Shimadzu AA-6880, Shimadzu, Tokyo, Japan). For each biological replicate, two technical replicates were measured for cadmium concentration, with the mean value used for statistical analysis [30].

It should be noted that hydroponic conditions differ from field soil environments in terms of cadmium bioavailability and root–soil interactions. Therefore, the cadmium accumulation observed in this study may not directly translate to paddy field conditions. Nevertheless, the relative differences in cadmium accumulation between varieties observed under the controlled hydroponic conditions provide useful information for screening low-cadmium rice germplasm in a standardized environment [31].

### 2.8. Statistical Analysis

All data are presented as mean ± standard deviation (SD). Statistical comparisons between the two varieties under the same temperature, organ, and time point were conducted using a one-tailed Welch’s *t*-test (assuming unequal variances), with the alternative hypothesis that the mean Cd content of Xiangwanxian 12 is lower than that of Yuzhenxiang. A *p*-value < 0.05 was considered statistically significant, with significance levels indicated as * *p* < 0.05, ** *p* < 0.01, and *** *p* < 0.001. All statistical analyses and graph preparation were performed using R software (version 4.3.2; R Core Team, 2023), specifically the base *t*-test function, and Origin 2018 (OriginLab Corp., Northampton, MA, USA).

## 3. Results

### 3.1. Primer Design

#### 3.1.1. LAMP Primer Design

**Figure 1:**
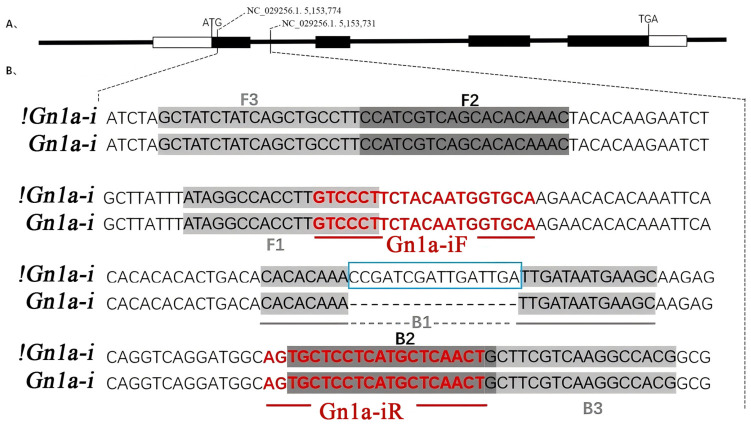
Structure of the *Gn1a* gene and positions of primers and restriction endonucleases on the target sequence (**A**) Structure of the *Gn1a* gene. ATG indicates the position of the start codon; TGA indicates the position of the stop codon; the white box before ATG represents the 5′UTR, and the white box after TGA represents the 3′UTR; black boxes represent exons. (**B**) *Gn1a-i*: represents the sequence with the *Gn1a-i* genotype; !*Gn1a-i*: represents the sequence without the *Gn1a-i* genotype; F3, F2, F1, B3, B2, B1 (gray background) indicate the positions of the six regions corresponding to the LAMP primers; Gn1a-1F and Gn1a-1R (red font) indicate the positions of the upstream and downstream primers for the InDel, and the blue box in the middle indicates the 16bp deletion; NC_029256.1. 5,153,774 indicates that the base “A” is at position 5,153,774 of GenBank No. NC_029256.1, and NC_029256.1. 5,153,731 indicates that the base “G” is at position 5,153,731 of GenBank No. NC_029256.1.

A set of LAMP primers targeting the *Gn1a-i* allele was designed using PrimerExplorer V5 based on the six characteristic regions of the target sequence (Figure 1; Table 1). The primer set specifically targeted the 16 bp deletion located in the 5′ untranslated region (5′UTR) of the *Gn1a* gene. To enhance allele specificity, part of the B1 region of the inner primer BIP was designed to overlap the deletion site. This design ensured that efficient amplification would occur only when the target sequence containing the *Gn1a-i* allele was present. In contrast, the absence of the deletion resulted in primer–template mismatches that significantly reduced amplification efficiency.

In addition, the 5′ ends of primers F3 and FIP were labeled with 6-FAM and biotin, respectively (Table 1), allowing the LAMP products to be detected rapidly using a lateral flow dipstick (LFD) system. This dual-labeling strategy enabled visual detection of amplification products through antigen–antibody interactions on the LFD strip, thereby facilitating rapid screening of rice varieties carrying the *Gn1a-i* allele.

#### 3.1.2. PCR Primer Design

To verify the reliability of the LAMP assay, a pair of PCR primers (Gn1a-1F and Gn1a-1R) targeting the insertion–deletion (InDel) marker specific to the *Gn1a-i* allele was designed using Primer3. The primers were located in the conserved sequences flanking the 16 bp deletion in the 5′UTR region of the *Gn1a* gene (Figure 1; Table 1).

Because the primers flank the deletion site, the presence or absence of the 16 bp deletion results in different amplification fragment sizes. Rice varieties carrying the *Gn1a-i* allele produce a PCR fragment of 108 bp, whereas varieties lacking the deletion generate a fragment of 124 bp. This fragment length polymorphism enables clear discrimination of the *Gn1a-i* allele by agarose gel electrophoresis and provides a reliable reference method for validating the LAMP screening results.

### 3.2. LAMP Assay Screening

**Figure 2:**
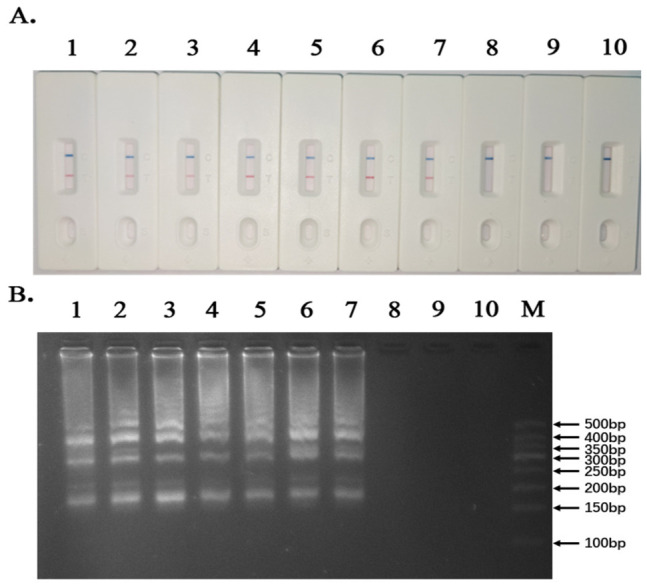
LAMP Detection Results. (**A**) Electrophoretic detection of LAMP products. (**B**) LFD detection results. 1–10 are: Xiangwanxian 12, 9311, Zhenshan 97, N22, R498, R600, Jiayu 253, Zhonghua 11, Nipponbare, CG14; M is 100bp DNA Ladder.

The designed LAMP primer set was used to screen ten rice varieties: Xiangwanxian 12, 9311, Zhenshan 97, N22, R498, R600, Jiayu 253, Zhonghua 11, Nipponbare, and CG14. Amplification products were analyzed by both lateral flow dipstick (LFD) detection and agarose gel electrophoresis (Figure 2).

The LFD results showed that Xiangwanxian 12, 9311, Zhenshan 97, N22, R498, R600, and Jiayu 253 produced two visible bands (test line and control line), indicating positive amplification of the *Gn1a-i* allele. In contrast, Zhonghua 11, Nipponbare, and CG14 displayed only a single control band, indicating the absence of the *Gn1a-i* allele (Figure 2).

The agarose gel electrophoresis results were consistent with the LFD detection results. Ladder-like amplification patterns characteristic of LAMP products were observed in Xiangwanxian 12, 9311, Zhenshan 97, N22, R498, R600, and Jiayu 253, whereas no ladder-like bands were detected in Zhonghua 11, Nipponbare, or CG14 (Figure 2). Taken together, these results demonstrated that seven rice varieties, namely Xiangwanxian 12, 9311, Zhenshan 97, N22, R498, R600, and Jiayu 253 carry the *Gn1a-i* allele, while Zhonghua 11, Nipponbare, and CG14 do not.

### 3.3. PCR Validation of the LAMP Screening Results

**Figure 3:**
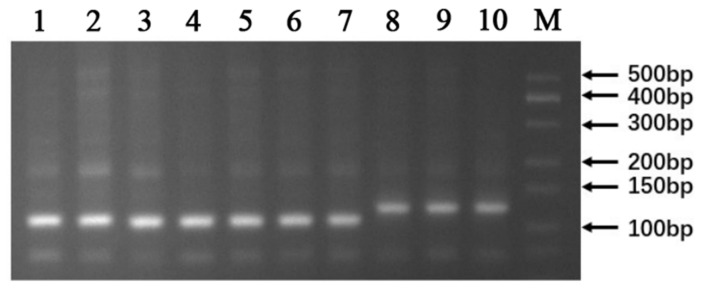
PCR Detection Results.Lane 1–10 are: Xiangwanxian 12, 9311, Zhenshan 97, N22, R498, R600, Jiayu 253, Zhonghua 11, Nipponbare, CG14; M is the DNA Ladder.

To validate the reliability of the LAMP screening results, PCR amplification was performed using the InDel marker primers Gn1a-1F and Gn1a-1R on the same ten rice varieties. The PCR products were analyzed by agarose gel electrophoresis (Figure 3). The results showed that Xiangwanxian 12, 9311, Zhenshan 97, N22, R498, R600, and Jiayu 253 produced amplification fragments of 108 bp, corresponding to the *Gn1a-i* allele containing the 16 bp deletion. In contrast, Zhonghua 11, Nipponbare, and CG14 produced amplification fragments of 124 bp, indicating the absence of the deletion. These PCR results were fully consistent with the LAMP assay results, confirming the accuracy and reliability of the LAMP-based screening method for detecting the *Gn1a-i* allele in rice varieties.

### 3.4. Evaluation of Cadmium Accumulation Characteristics

Based on the LAMP screening results, the rice variety Xiangwanxian 12 carrying the Gn1a-i allele was selected for further analysis, while the high-cadmium-accumulating variety Yuzhenxiang was used as the control. The evaluation of cadmium accumulation characteristics was conducted as described in Section 2.7. Seedlings of the two rice varieties were exposed to cadmium stress under three temperature gradients (low, medium, and high) for six consecutive days. Fresh weight, dry weight, and cadmium concentrations in roots, stem sheath, and leaves were measured at different sampling times.

**Figure 4:**
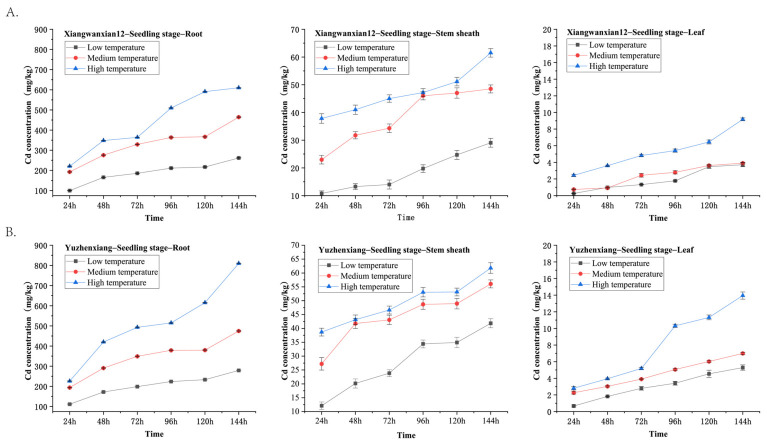
Cadmium levels in roots, stem sheath, and leaves of (**A**) Xiangwanxian 12 and (**B**) Yuzhenxiang under low (15–20 °C), medium (22–27 °C), and high (30–35 °C) temperature treatments over 144 h. Data are presented as mean ± SD (*n* = 3).

The experimental results showed that cadmium concentrations in roots, stem sheath, and leaves increased over the treatment duration, corresponding to rising temperatures in both varieties (Figure 4). Under all temperature conditions, cadmium accumulation followed the pattern root > stem sheath > leaf. Although temperature did not alter this relative distribution, it significantly enhanced cadmium uptake, with the highest accumulation observed under high-temperature conditions. Notably, Xiangwanxian 12 consistently exhibited lower cadmium concentrations than Yuzhenxiang across all organs, time points, and temperature conditions.

**Figure 5:**
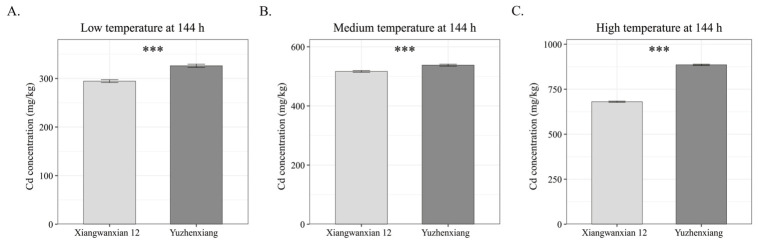
Sum of cadmium contents in roots, stem sheath, and leaves of Xiangwanxian 12 and Yuzhenxiang at 144 h under three temperature treatments. Data are presented as mean ± SD (*n* = 3). (**A**) Low temperature (15–20 °C), (**B**) medium temperature (22–27 °C), (**C**) high temperature (30–35 °C). Asterisks (***) indicate significant differences between the two varieties (one-tailed *t*-test, *p* < 0.01 for all three temperatures). Gray-scale columns: dark gray, Yuzhenxiang; light gray, Xiangwanxian 12.

To further compare the overall cadmium accumulation capacity of the two varieties, cadmium contents in roots, stem sheath, and leaves at 144 h were measured and compiled across all temperature treatments. As shown in Figure 5, the total cadmium content in the measured organs of Xiangwanxian 12 was significantly lower than that of Yuzhenxiang (one-tailed *t*-test, *p* < 0.01) under all conditions. The reductions were 9.6%, 4.0%, and 23.2% under low-, medium-, and high-temperature treatments, respectively. These results indicate that Xiangwanxian 12 exhibits a lower cadmium accumulation capacity than the high-cadmium-accumulating variety Yuzhenxiang, suggesting that it represents a promising germplasm resource for the development of low-cadmium rice varieties.

## 4. Discussion

Ensuring both high yield and food safety in rice production has become increasingly important in the context of global population growth and environmental contamination. According to recent reports from the World Food Programme, hundreds of millions of people worldwide continue to experience severe food insecurity, while a considerable proportion of cultivated land has been affected by heavy metal contamination [32,33,34]. In particular, cadmium (Cd) contamination of paddy soils has emerged as a major agricultural and public health concern because Cd can be readily absorbed by rice plants and subsequently enter the human food chain [35]. Furthermore, compared with conventional remediation technologies for heavy metal–contaminated farmland, which are often costly and time-consuming [36,37], the cultivation of low-cadmium-accumulating crop varieties represents a practical and sustainable strategy. By selecting appropriate rice varieties for contaminated soils, cadmium accumulation in rice grains can be minimized, thereby enabling the safe utilization of moderately and lightly cadmium-contaminated farmland that would otherwise be abandoned [38].

In this study, a rapid molecular screening method targeting the *Gn1a-i* allele was developed using loop-mediated isothermal amplification (LAMP) combined with lateral flow dipstick (LFD) detection (Figure 2). Compared with conventional PCR-based methods, the LAMP–LFD system provides several advantages, including high sensitivity, rapid amplification, and visual detection without the need for complex laboratory equipment. The results from ten rice varieties demonstrated that the designed LAMP primer set (Figure 1; Table 1) could specifically detect the *Gn1a-i* allele, and the screening results were fully consistent with PCR validation (Figure 3). These findings indicate that the LAMP-based method is reliable and suitable for rapid identification of rice germplasm carrying the high-yield *Gn1a-i* allele. While PCR-based markers for *Gn1a* detection are well established, the method typically requires thermal cycling and gel electrophoresis, which are costly and inconvenient, particularly for small-scale farmers [39,40]. In contrast, the LAMP-LFD system developed here enables isothermal amplification and visual readout within 60 min, offering a simpler, faster, and more cost-effective alternative. Using LAMP-LFD, ten varieties of rice were evaluated in this study, of which seven—Xiangwanxian 12, 9311, Zhenshan 97, N22, R498, R600, and Jiayu 253—were found to carry the *Gn1a-i* allele, whereas Zhonghua 11, Nipponbare, and CG14 did not.

Temperature is an important environmental factor influencing heavy metal uptake and transport in plants [41]. In this study, cadmium accumulation in Xiangwanxian 12 and Yuzhenxiang were investigated under different temperatures. The results show that cadmium accumulation increased with both treatment duration and temperature (Figure 4). This phenomenon may be related to the increased metabolic activity of plant roots at elevated temperatures, which can promote ion transport processes and enhance the uptake of heavy metals from the surrounding environment [42]. In addition, temperature-induced changes in membrane permeability and transporter activity may further facilitate cadmium absorption and translocation within rice plants [43]. At the molecular level, temperature was reported to influence the expression of key transporter genes, such as *OsNramp5* for Cd uptake and *OsHMA2* for long-distance transport [44].

The distribution pattern of cadmium among plant organs observed in this study, with the highest concentrations in roots followed by stem sheath and the lowest in leaves (Figure 4), is consistent with previous studies on cadmium transport in rice [45]. Roots typically serve as the primary site of cadmium accumulation because they are directly exposed to contaminated soil or nutrient solution. Only a portion of the absorbed cadmium is translocated to aboveground tissues through the xylem, resulting in lower cadmium concentrations in stems and leaves [46]. This physiological barrier plays an important role in limiting the transport of cadmium to edible plant parts.

Most importantly, comparative analysis between the two tested cultivars revealed significant differences in cadmium accumulation capacity. Under identical experimental conditions, cadmium concentrations in all examined organs of Yuzhenxiang were consistently higher than those in Xiangwanxian 12. These results indicate that Xiangwanxian 12 exhibits relatively low cadmium accumulation compared with the high-cadmium-accumulating cultivar Yuzhenxiang. Considering that Xiangwanxian 12 also carries the *Gn1a-i* allele associated with increased grain number per panicle [15], this variety represents a promising germplasm resource for breeding rice cultivars with both high yield potential and reduced cadmium accumulation.

Some limitations should be acknowledged in this study. First, only ten rice varieties were screened, and one variety (Xiangwanxian 12) carrying the *Gn1a-i* allele and exhibiting low cadmium accumulation was ultimately identified. Second, all experiments were conducted under hydroponic conditions, which may not fully reflect the complex environment of field paddy environments. Nevertheless, a reliable screening system was established and can be readily applied to larger-scale germplasm evaluation. Moreover, the controlled hydroponic system allows precise regulation of cadmium concentration and temperature, and the significant differences observed between varieties under standardized conditions provide a robust basis for identifying low-cadmium rice cultivars [31]. These findings lay a foundation for subsequent field validation and practical applications.

## 5. Conclusions

In conclusion, this study successfully developed a LAMP–LFD method for the rapid screening of the Gn1a-i allele. Using this approach, Xiangwanxian 12 was identified as a rice variety carrying the high-yield allele and exhibiting significantly lower cadmium accumulation than the high-cadmium-accumulating variety Yuzhenxiang under all temperature treatments. These findings highlight the potential of Xiangwanxian 12 as a valuable germplasm resource for breeding high-yield, low-cadmium rice cultivars. The established screening method provides a reliable tool for germplasm evaluation and is expected to facilitate the safe utilization of cadmium-contaminated farmland. Future research should include larger-scale field trials of Xiangwanxian 12, as well as expanded germplasm screening using this method to identify additional rice varieties that carry the Gn1a-i allele and exhibit low cadmium accumulation.

## Figures and Tables

**Table 1:** Primer information.

Primers	Sequences (5′~3′)	Usage
F3	(6-FAM)-GCTATCTATCAGCTGCCTT	Forward Outer Primer for LAMP
FIP	(biotin)-AGGGACAAGGTGGCCTATCCATCGTCAGCACACAAAC	Forward Inner Primer for LAMP
B3	CGTGGCCTTGACGAAG	Backward Outer Primer for LAMP
BIP	CACACAAATTGATAATGAAGCCAGTTGAGCATGAGGAGCA	Backward Inner Primer for LAMP
Gn1a-iF	GTCCCTTCTACAATGGTGCA	Forward Primer for InDel
Gn1a-iR	AGTTGAGCATGAGGAGCACT	Backward Primer for InDel

## Data Availability

Additional data are available from the corresponding authors upon request.
